# Inhibitory activity of apogossypol in human prostate cancer *in vitro* and *in vivo*

**DOI:** 10.3892/mmr.2015.3326

**Published:** 2015-02-10

**Authors:** WENHUA ZHAN, XINGBIN HU, JING YI, QUNXING AN, XIAOFENG HUANG

**Affiliations:** 1 Department of Radiotherapy, The General Hospital of Ningxia Medical University, Yinchuan, Ningxia 750004, P.R. China; 2 Department of Blood Transfusion, Xijing Hospital, Fourth Military Medical University, Xi’an, Shaanxi 710032, P.R. China; 3 Central Laboratory, Fourth Military Medical University, Xi’an, Shaanxi 710032, P.R. China

**Keywords:** apogossypol, gossypol, apoptosis, prostate cancer

## Abstract

Apogossypol, a gossypol derivative, is a novel small-molecule inhibitor of the Bcl-2 family proteins and has been demonstrated to have anti-tumor activities. Prostate cancer is the most common malignancy in males, for which chemotherapy is the usual treatment option in clinical practice. The aim of the present study was to investigate the growth inhibitory effects of apogossypol on prostate cancers *in vitro* and *in vivo*. An MTT assay and a colony formation assay were used to assess the anti-survival and anti-proliferation effects of apogossypol in LNCaP cells. Immunofluorescence was performed in order to detect the expression levels of apoptosis-associated proteins in xenograft tumors following apogossypol treatment. Apogossypol exerted strong anti-tumor effects on LNCaP cells in a dose-dependent manner. Furthermore, immunofluorescence revealed that apogossypol inhibited the growth and proliferation of prostate cancer cells by downregulating Bcl-2 protein expression and activating caspase-3 and -8. In addition, the *in vivo* study indicated that apogossypol significantly inhibited tumor growth in a dose-dependent manner with reduced toxicity compared with gossypol. In conclusion, the present study indicated that apogossypol effectively inhibited the growth and proliferation of prostate cancer cells and may be a potential agent for prostate cancer therapy.

## Introduction

Prostate cancer is one of the most common malignancies and the second leading cause of mortality among male cancer patients in developed countries (1). Due to the limited treatments available for this disease, although chemotherapy and radiotherapy are currently used, and poor prognosis and therapy failure in prostate cancers usually occur (2,3). One of the main reasons is that the growth and survival of prostate cancer cells depend on androgens, which are also associated with tumor regression (4). At present, no effective treatments prostate cancers are available, and therefore, it is important to identify novel chemotherapeutic drugs and to develop effective treatment strategies for prostate cancer.

Apoptosis has an important role in keeping the balance between cell proliferation and death in normal tissues (5). B-cell lymphoma 2 (Bcl-2) family proteins regulate apoptosis in cancer progression, and they have been divided into pro- and anti-apoptotic groups, including Bcl-2, Bcl-extra large (xL), myeloid leukemia cell differentiation protein (Mcl-1), and Bcl-2 family proteins 1, 2 and 10 (6,7). It is noteworthy that the anti-apoptotic Bcl-2 family proteins are actively involved in various cancer types in humans, making them attractive targets for developing novel anti-cancer drugs (8,9). Additionally, structural studies reveal that the anti-apoptotic Bcl-2 protein has a hydrophobic groove (10,11), and it could form a binding pocket for the pro-apoptotic members with Bcl-2 homology domain 3 (BH3) domains, interfering with their pro-apoptotic functions (12). Thus, targeting the binding pocket of anti-apoptotic Bcl-2 proteins is a new and promising strategy for cancer therapy and drug discovery (13).

**Figure 1:**
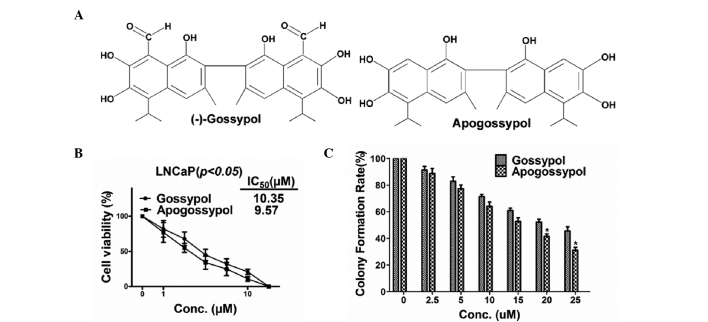
Apogossypol inhibits the survival and the proliferation of LNCaP cells. (A) Chemical structures of gossypol and apogossypol. (B) An MTT assay was used to evaluate the *in vitro* anti-survival effects of apogossypol and gossypol. (C) A colony formation assay was performed to assess the anti-proliferation effects of apogossypol and gossypol. ^*^P<0.05, compared with the gossypol treated group.

Recently, several non-peptide small molecule inhibitors of Bcl-2 family proteins have been synthesized and extracted (14). Gossypol, a polyphenolic compound isolated from cotton seeds and roots, is one of these effective anti-tumor drugs undergoing evaluation in pre-clinical trials, which has been reported to have anti-proliferation activities against several types of cancer *in vitro* and anti-tumor effects *in vivo* (15,16). This natural product is also a potent and effective male contraceptive drug in an early clinical stage (17–19). However, the two aldehyde groups in gossypol are associated with toxicity and potential non-specific activities (20). Therefore, it is imperative to develop novel gossypol derivatives with a higher binding affinity to Bcl‑2 proteins as well as good selectivity between normal and cancer cells with varying levels of Bcl-2 proteins (21). Researchers are synthesizing novel gossypol derivatives in order to optimize its chemical structure and improve its anti-cancer effect by removing aldehyde groups, to achieve superior anti-proliferation activity with less toxicity in nasopharyngeal carcinoma, prostate cancer, human leukemic monocyte lymphoma, diffuse large-cell lymphoma, follicular lymphoma, pancreatic cancer cells and human hepatocellular carcinoma (22,23). The derivative apogossypolone has been synthesized and its anti-cancer effects have been investigated. The results revealed that apogossypolone effectively inhibited the growth and proliferation of gastric and prostate cancer cell lines *in vitro* and *in vivo* (24,25). In addition, our group and others have designed and synthesized apogossypol (Fig. 1A), a novel gossypol derivative lacking two aldehyde groups, which retains the activity against the anti-apoptotic Bcl-2 family proteins *in vitro* (26). Based on the chemical design, apogossypol was expected to exert significantly lower toxicity while maintaining a similar anti-cancer activity to that of gossypol. However, whether or not apogossypol could actually inhibit the growth and proliferation of prostate cancer cells has yet to be established. In the present study, the inhibitory effects of apogossypol on human prostate cancers were investigated in order to demonstrate and compare the anti‑cancer efficiencies between apogossypol and gossypol on prostate cancers *in vitro* and *in vivo*.

## Materials and methods

### Cell lines and reagents

The LNCaP human prostate cancer cell line was purchased from the American Type Culture Collection (Manassas, VA, USA). The cells were cultured in RPMI1640 medium (Gibco-BRL, Grand Island, NY, USA) supplemented with 10% fetal bovine serum (FBS; Gibco-BRL) and 1% penicillin/streptomycin in a humidified incubator at 37°C with 5% CO_2_. Apogossypol and gossypol were synthesized and extracted in our laboratory (25), dissolved in dimethyl sulfoxide (DMSO) and stored at ‑20°C. Working solutions were prepared by diluting the stock solution with culture medium before use. MTT was purchased from Sigma-Aldrich (St. Louis, MO, USA). The anti-Bcl-2, anti-caspase-3, and anti-caspase-8 antibodies were purchased from Maixin Biotechnology (Fuzhou, China), Zhongshan Golden Bridge Biotechnology (Beijing, China) and Boster Biological Engineering (Wuhan, China), respectively. Monkey anti-mouse immunoglobulin (Ig)G labeled with fluorescein isothiocyanate (FITC) and goat anti-rabbit IgG labeled with rhodamine were purchased from Santa Cruz Biotechnology (Santa Cruz, CA, USA).

### MTT assay

The cytotoxic effect of apogossypol and gossypol on prostate cancer cell lines was measured by the MTT assay. LNCaP cells were seeded onto sterile 96‑well flat‑bottomed plates and incubated overnight. Then diluted apogossypol and gossypol were added into each well with gradient concentrations (2–20 *μ*mol/l). For the cell viability test, tumor cells were suspended in a mixed solution of 200 *μ*l complete medium and 0.2 *μ*l DMSO, and wells with 200 *μ*l complete medium were used as blank controls. The plates were incubated at 37°C with 5% CO_2_ for 72 h. The medium was then removed, and 0.5 *μ*mol/l MTT was added into the wells. After another 4 h, 150 *μ*l DMSO was added into each well. The absorbance was read at 570 nm on a microplate reader (SpectraMax^®^ M2, Molecular Devices, Sunnyvale, CA, USA). The drug concentration yielding 50% cell inhibition (IC_50_) was determined. All experiments were performed in triplicate.

### Colony formation assay

The colony formation assay was conducted on LNCaP cells. The cells were seeded in six-well plates at a density of 200/well. Apogossypol and gossypol were added 24 h later at appropriate doses. Following five days of incubation, 0.5 ml serum was added into each well. The colonies were stained with crystal violet on day 14 and the colonies consisting of >50 cells were counted.

### Terminal deoxynucleotidyl transferase dUTP nick end labeling (TUNEL) assay

To assess apoptosis in the tumors, the TUNEL assay was carried out using an In Situ Cell Death Detection Kit (Boehringer-Mannheim, Mannheim, Germany). Briefly, paraffin‑embedded tissue sections were treated with proteinase K (20 *μ*g/ml) in 10 mmol/l Tris-HCl (pH 7.5) for 30 min at room temperature and afterwards they were dewaxed and rehydrated. The slides were rinsed with phosphate-buffered saline (PBS) twice, for 5 min each time. The sections were then incubated with 50 *μ*l TUNEL reaction mixture at 37°C for 1 h in a humidified chamber. Following incubation, the slides were rinsed with PBS three times, for 5 min each time, and the apoptotic cells were visualized with an Olympus FV1000 laser scanning confocal microscope (Olympus, Tokyo, Japan). A positive control was prepared by treating the samples with DNase I prior to TUNEL staining.

### Animal experiments

Female athymic nude (nu/nu) mice (4–6 weeks of age, weighing 20–25 g) were purchased from the animal center of the Fourth Military Medical University. All animal experiments were performed according to the protocol approved by the Fourth Military Medical University Guidelines for the Use and Care of Animals. LNCaP cells (2×10^6^) were injected subcutaneously into each mouse. The tumor volume was measured every two days using a caliper and calculated according to the following formula: Tumor volume=L×W^2^, where L and W were the length and width, respectively (24). When subcutaneous tumor sizes reached 150–200 mm^3^, these mice were randomly divided into three groups, each group consisting of 10 mice. Next, they were treated with apogossypol and gossypol, respectively, at 20 mg/kg intraperitoneally, q.d. every 7 d for 28 d. The vehicle control group received the same amount of DMSO as in the treatment groups. The tumor volume was detected every day. The tumor tissues were fixed in 10% formalin solution. The tissues were embedded with paraffin, and the sections were prepared. Samples were stained with hematoxylin and eosin (H&E) and microscopically examined (Olympus IX81; Olympus, Tokyo, Japan).

### Immunofluorescence

The tumor tissues were dissected, fixed with formaldehyde (40 *μ*g/ml), embedded in paraffin and deparaffinized with xylene. Following washing with water, the sections were blocked with 250 *μ*l/ml goat serum for 30 min. Next, mouse anti-human Bcl-2 monoclonal antibody (1:50 dilution), rabbit anti-caspase-3 polyclonal antibody (1:100 dilution) or rabbit anti-caspase-8 polyclonal antibody (1:100 dilution) was separately used for incubation at 4°C overnight in a humidified chamber. Following washing with PBS for three times, monkey anti-mouse IgG labeled with FITC or goat anti-rabbit IgG labeled with rhodamine (1:100 dilution) was added and the samples were incubated at 37°C for another 1 h. After washing with PBS for three times, the samples were mounted with glycerol buffer and examined under a microscope (Olympus IX81). In total, 10 high-power fields per section were observed.

### Statistical analysis

Data were presented as the mean ± standard deviation. All the statistical analysis was performed with SPSS 16.0 software (Chicago, IL, USA). Student’s t-test was used for statistical comparisons between groups. P<0.05 was used to indicate a statistically significant difference.

## Results

### Apogossypol inhibits the survival of LNCaP cells

To investigate the inhibitory effects of apogossypol and gossypol on LNCaP cell survival, the MTT assay was performed. The results demonstrated that apogossypol inhibited the proliferation of LNCaP cells in a time- and dose-dependent manner, in a similar way with gossypol (Fig. 1B). The concentration for 50% inhibition (IC_50_) on LNCaP cells within ~72 h was 9.57 *μ*mol/l, while the IC_50_ of gossypol on LNCaP cells was 10.35 *μ*mol/l. The inhibitory activities of apogossypol and gossypol at the same drug concentration were not significantly different from each other, suggesting that the removal of two aldehyde groups had little effect on the anti-tumor effect of gossypol.

### Apogossypol inhibits the proliferation of prostate cancer cells

To further evaluate the anti-tumor effects of apogossypol and gossypol, the colony formation assay was performed. The treatment groups were treated with different drug concentrations of apogossypol or gossypol, and the control group was treated with DMSO. After 14 days of treatment, the percentage of colony formation was calculated. Apogossypol potently inhibited the colony formation of LNCaP cells (Fig. 1C). Compared with the vehicle control, 15 *μ*mol/l apogossypol inhibited >56% of the colony formation, while 25 *μ*mol/l gossypol inhibited >45% of the colony formation. These data indicated that apogossypol and gossypol had strong anti-tumor activities. Furthermore, the results also indicated that the anti-tumor activity of apogossypol was slightly stronger compared to that of gossypol, which was in accordance with the result of the MTT assay.

### Apogossypol induces apoptosis in LNCaP cells

**Figure 2:**
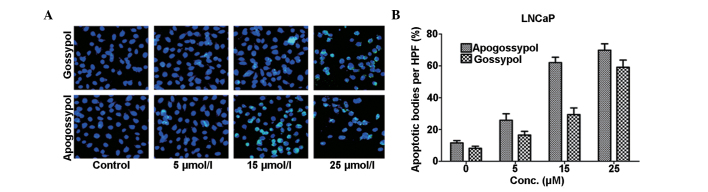
Apogossypol induces apoptosis in LNCaP cells. (A) LNCaP cells were incubated with either apogossypol or gossypol at the indicated concentrations, or DMSO, for 48 h. Next, Hoechst 33258 staining was performed to detect apoptotic cells (cyan), while the nuclei in normal cells were stained with DAPI (blue) (magnification, ×400). (B) Statistical analysis of apoptotic cells with either apogossypol or gossypol. The histogram represents the percentage of apoptotic cells among 200 cells within a high‑power field. Values are expressed as the mean ± standard deviation from three independent experiments. ^**^P<0.01 compared with the gossypol treated group. HPF, high‑power field; DAPI, 4′,6-diamidino-2-phenylindole; DMSO, dimethylsulfoxide.

Hoechst 33258 staining was performed next to evaluate the levels of apoptosis in different drug concentration groups. The cells with changed nuclear morphologies indicated by Hoechst 33258 staining (cyan) were TUNEL-positive cells, while the nuclei in normal cells were stained with DAPI (blue). The cells treated with high drug concentrations exhibited evident apoptotic characteristics, including shrinkage and nuclear fragmentation (Fig. 2A). Compared with the gossypol group, it was notable that there was an evident increase in the number of TUNEL-positive LNCaP cells following treatment with apogossypol at a concentration of 15 *μ*mol/l. The apoptotic rate following gossypol treatment was 24%, which was significantly lower than 62% following apogossypol treatment (P<0.01; Fig. 2B). The results indicated that inducing cell apoptosis may be a possible mechanism underlying the anti-tumor activity of apogossypol in prostate cancer.

### Apogossypol alters the expression levels of Bcl‑2, caspase‑3 and ‑8 in prostate tumors

**Figure 3:**
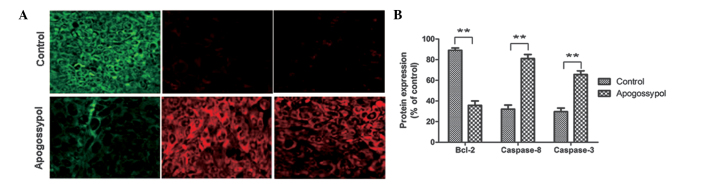
Apogossypol alters the expression levels of Bcl‑2, caspase‑3, and caspase‑8 in prostate tumors. (A) Immunofluorescence was performed to detect the expression levels of Bcl‑2, caspase‑3, and caspase‑8 in LNCaP xenografts treated with apogossypol. Control group was treated with DMSO (magnification, ×400). (B) Statistical analysis of protein expression levels in LNCaP cells. The histogram represents the relative expression levels of Bcl-2, caspase-3, and caspase-8, compared with non-treated cells. Values expressed as mean ± standard deviation from three independent experiments. ^**^P<0.01 compared with the control group. DMSO, dimethyl sulfoxide; Bcl-2, B-cell lymphoma 2.

To investigate the possible molecular mechanism(s) through which apogossypol triggered apoptosis, immunofluorescence was performed to observe the changes of the protein expression levels of the Bcl-2 family members in LNCaP xenograft cells treated with apogossypol. An evidently high percentage of tumor cells expressed Bcl-2 in the control group, while apogossypol treatment caused a significant decrease in Bcl-2 expression levels in tumor tissues, indicating that apogossypol induced apoptosis in the LNCaP xenograft tumor (Fig. 3A). In addition, the expression levels of caspase-3 and -8 were weak in the control group, while they were strong in the groups treated with apogossypol. Compared with the control group, it was notable that there was a clear increase in the protein expression levels in LNCaP cells treated with 25 *μ*mol/l apogossypol. The relative protein expression levels of Bcl-2 were only 37% in cells treated with apogossypol, compared with 84% in the control group (P<0.01; Fig. 3B). However, the relative expression levels of caspase-3 and caspase-8 in the apogossypol group were 67 and 81%, respectively, which were both significantly higher compared to those in the control group (P<0.01). These findings indicated that apogossypol induced tumor cell apoptosis by downregulating the Bcl-2 protein expression levels and upregulating caspase-3 and -8 expression levels.

### Apogossypol reduces tumor growth of LNCaP xenografts in vivo

**Figure 4:**
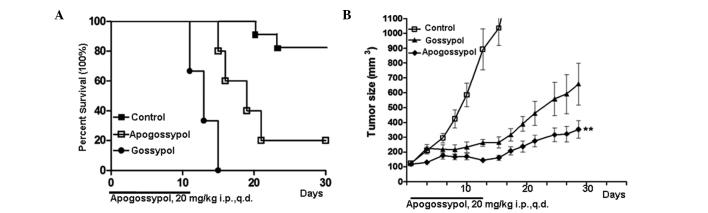
Apogossypol reduces tumor growth of LNCaP xenografts *in vivo*. LNCaP cells were inoculated into mice through subcutaneous injection. When subcutaneous tumor masses developed to ~70–100 mm^3^, the mice were treated with or without 100 mg kg^−1^ apogossypol every 7 d for 28 d by intraperitoneal injection. (A) Survival rates of mice bearing LNCaP cell xenografts, treated with apogossypol or gossypol. (B) The tumor volumes were measured by caliper in mice bearing LNCaP cell xenografts, treated with apogossypol or gossypol. Data are expressed as mean ± standard deviation from three independent experiments. ^**^P<0.01 compared with the control group.

**Figure 5:**
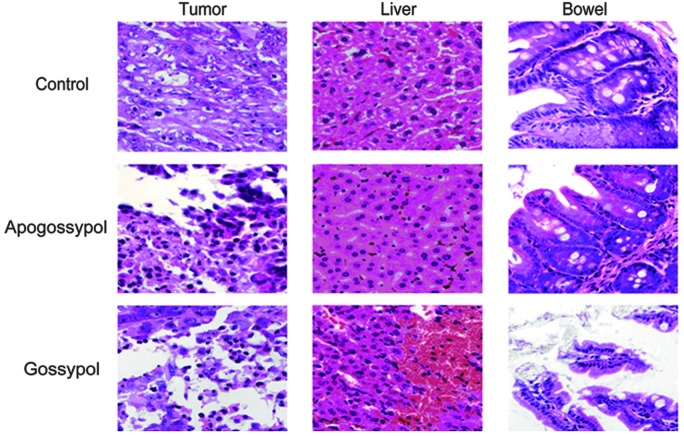
Pathological analysis of tumor and normal tissues. H&E staining was performed to observe the pathological changes. Mice in the control, apogossypol and gossypol groups were sacrificed, and the tumor and normal tissues were removed and fixed. The sections were stained with H&E and microscopically examined. Representative H&E staining results are shown (magnification, ×400). Compared with gossypol, apogossypol showed lower toxicity to normal tissue. H&E, hematoxylin and eosin.

Due to its modified structure, apogossypol was expected to exhibit lower toxicity while maintaining the significant anti-growth and anti-tumor activities *in vitro*, similar to those of gossypol. Therefore, the present study further evaluated the anti-cancer effect of apogossypol in mice bearing subcutaneous LNCaP cell xenografts. The tumor growth was monitored and measured by a caliper and balance. The survival rate of the mice was notably improved by apogossypol (Fig. 4). Of note, the tumor sizes were also markedly decreased by apogossypol treatment (P<0.01). These results indicated that apogossypol had significant anti-cancer activity in prostate cancers *in vivo*, compared with gossypol at the same drug concentration (20 mg kg^−1^). Finally, the pathological examination was carried out by H&E staining. The cells in tumor tissues of the apogossypol treatment group exhibited necrotic or pyknotic nuclei (Fig. 5). However, there were no evident lesions in other normal tissues. The results demonstrated that apogossypol produced an excellent anti-cancer therapeutic response, while having low toxicity to normal tissues.

## Discussion

Although chemotherapy and radiotherapy are currently common treatments for prostate cancers, limited therapeutic methods are available for the disease (27). Thus, identifying novel chemotherapeutic drugs or developing effective treatment strategies is important for disease management (28). Recently, the strategy of blocking anti-apoptotic protein activities has gained increasing attention. Several non-peptide small molecular inhibitors of Bcl-2 family proteins have been synthesized and used in studies for therapies against various types of cancers (29,30). However, the effects of apogossypol in prostate cancer therapy have never been established. To the best of our knowledge, proliferation and apoptosis are extensively used biomarkers for diagnosis and measurement of tumor aggressiveness, which thereby contribute to evaluating the tumor responses to novel anti-cancer drugs (31,32). Thus, the anti-proliferation and apoptosis-inducing effects of apogossypol in prostate cancers were evaluated *in vitro*. The MTT and colony formation assays revealed that apogossypol effectively inhibited cell growth and induced evident apoptosis in prostate cancer cells. The anti-growth effects and apoptosis-inducing abilities between apogossypol and gossypol were further compared. Based on these results, it is reasonable to postulate that the removal of two aldehyde groups may have no effect on the *in vitro* growth inhibition ability of gossypol. In addition, several previous studies have reported that gossypol has a synergistic effect in enhancing anti-cancer therapies (33,34). Therefore, it is hypothesized that apogossypol may be used as a safe and effective agent in combination with other targeting or conventional drugs for therapy of prostate cancers, which is now actively underway in our laboratory.

To facilitate the translation of apogossypol from research into clinical practice for prostate cancer therapy, the *in vivo* response to drug therapy must be addressed. The two aldehyde groups in the chemical structure of gossypol are associated with toxicity (35,36). Thus, apogossypol was synthesized by removing the two aldehyde groups and has been found to maintain the anti-cancer effects for several types of cancers, while exhibiting reduced toxicity (37,38). In the present study, the toxicities and tumor-inhibiting activities between apogossypol and gossypol were compared in nude mouse xenografts. The results showed that apogossypol exhibited significantly lower toxicity and caused more significant reduction in tumor size compared to gossypol, which is consistent with previous reports. Therefore, the *in vivo* data further verified the fact that the removal of the two aldehyde groups did not affect the BH3, which creates a hydrophobic surface pocket that may be a binding groove for anti-tumor drugs (39). These results indicated that apogossypol may be a novel and useful anti-cancer agent for prostate cancer therapy.

The death receptor pathway, the mitochondrial pathway and endoplasmic reticulum stress-induced apoptosis are three common ways to induce apoptosis (40,41). Bcl-2 family proteins are regarded as the central regulators of the apoptotic process, which has been divided into two groups, pro-apoptotic proteins, including Bcl-2 homologous antagonist killer, Bcl-2-associated death promoter, Bcl-2-interacting killer and Bcl-2-like protein 11, as well as anti-apoptotic proteins, including Bcl-2, Bcl-xL and Mcl-1 (42,43). All of them have become hot spots to develop novel anti-cancer drugs (44,45). As a result, there are numerous newly designed and synthesized chemotherapeutic agents targeting the BH-3 domains of anti-apoptotic Bcl-2 members to induce apoptosis and inhibit the function of Bcl-2/Bcl-xL proteins, which may be useful in the management and treatment of cancers (46,47). In fact, the ratio between Bcl-2, caspase-3 and -8 may be used to determine whether cancer cells are undergoing apoptosis or not (14,48). Thus, the expression levels of these proteins were examined in the xenografts treated with apogossypol through immunofluorescence. Our results demonstrated that apogossypol could alter the expression levels of Bcl-2 family proteins, downregulate Bcl-2 expression levels and lead to the activation of apoptosis proteins, including caspase-3 and -8. Therefore, in prostate cancers, apogossypol could activate the mitochondrial signaling pathway to promote cell death.

In conclusion, it was demonstrated that a novel small-molecule inhibitor of the anti-apoptotic Bcl-2 family proteins, apogossypol, had significant anti‑tumor activity *in vitro* and *in vivo* in prostate cancers. Apogossypol may bind to Bcl-2 family proteins and prevent the binding of pro-apoptotic proteins with BH-3 domains, unleashing the pro-apoptotic proteins to induce the apoptotic response. The present study indicated that apogossypol may be a promising novel agent for prostate cancer therapy.
